# The impact of cytogenetics on duration of response and overall survival in patients with relapsed multiple myeloma (long‐term follow‐up results from BSBMT/UKMF Myeloma X Relapse [Intensive]): a randomised, open‐label, phase 3 trial

**DOI:** 10.1111/bjh.15782

**Published:** 2019-02-06

**Authors:** Gordon Cook, Kara‐Louise Royle, Sheila O'Connor, David A. Cairns, A. John Ashcroft, Cathy D. Williams, Anna Hockaday, Jamie D. Cavenagh, John A. Snowden, Debo Ademokun, Eleni Tholouli, Vivienne E. Andrews, Matthew Jenner, Christopher Parrish, Kwee Yong, Jim Cavet, Hannah Hunter, Jenny M. Bird, Guy Pratt, Mark T. Drayson, Julia M. Brown, Treen C. M. Morris, the National Cancer Research Institute Haemato‐oncology Clinical Studies Group

**Affiliations:** ^1^ Leeds Institute of Cancer and Pathology University of Leeds Leeds UK; ^2^ Clinical Trials Research Unit Leeds Institute of Clinical Trials Research University of Leeds Leeds UK; ^3^ HMDS Leeds Teaching Hospitals Trust Leeds UK; ^4^ Mid‐Yorkshire NHS Trust Wakefield UK; ^5^ Department of Haematology Centre for Clinical Haematology Nottingham City Hospitals Nottingham UK; ^6^ Department of Haematology Barts & The London NHS Trust London UK; ^7^ Department of Haematology Sheffield Teaching Hospitals NHS Foundation Trust Sheffield UK; ^8^ Ipswich Hospital NHS Trust Ipswich UK; ^9^ Department of Haematology Manchester Royal Infirmary Manchester UK; ^10^ Medway Maritime Hospital Kent UK; ^11^ University Hospital Southampton NHS Foundation Southampton UK; ^12^ Department of Haematology University College Hospital London UK; ^13^ Department of Haematology The Christie NHS Foundation Trust Manchester UK; ^14^ Department of Haematology Plymouth Hospitals Trust Plymouth UK; ^15^ Department of Haematology University Hospitals Bristol NHS Trust Bristol UK; ^16^ Department of Haematology Heart of England NHS Trust Birmingham UK; ^17^ University of Birmingham Birmingham UK; ^18^ Queen's University Belfast UK

**Keywords:** relapsed multiple myeloma, cytogenetics, duration of response, overall survival, salvage ASCT

## Abstract

The Myeloma X trial (ISCRTN60123120) registered patients with relapsed multiple myeloma. Participants were randomised between salvage autologous stem cell transplantation (ASCT) or weekly cyclophosphamide following re‐induction therapy. Cytogenetic analysis performed at trial registration defined t(4;14), t(14;16) and del(17p) as high‐risk. The effect of cytogenetics on time to progression (TTP) and overall survival was investigated. At 76 months median follow‐up, ASCT improved TTP compared to cyclophosphamide (19 months (95% confidence interval [95% CI] 16–26) vs. 11 months (9–12), hazard ratio [HR]: 0·40, 95% CI: 0·29–0·56, *P* < 0·001), on which the presence of any single high‐risk lesion had a detrimental impact [likelihood ratio test (LRT): *P* = 0·011]. ASCT also improved OS [67 months (95% CI 59‐not reached) vs. 55 months (44–67), HR: 0·64, 95% CI: 0·42–0·99, *P* = 0·0435], with evidence of a detrimental impact with *MYC* rearrangement (LRT:* P* = 0·021). Twenty‐one (24·7%) cyclophosphamide patients received an ASCT post‐trial, median OS was not reached (95% CI: 39‐not reached) for these participants compared to 31 months (22–39), in those who did not receive a post‐trial ASCT. The analysis further supports the benefit of salvage ASCT, which may still be beneficial after second relapse in surviving patients. There is evidence that this benefit reduces in cytogenetic high‐risk patients, highlighting the need for targeted study in this patient group.

Genomic landscape analysis in multiple myeloma (MM) has been recognized as a prognosis‐defining criterion for several decades, especially in newly diagnosed patients, utilising interphase fluorescence *in situ* hybridization (iFISH). In particular, mutation and deletions of the tumour‐suppressor gene, *TP53* on chromosome 17 (17p deletion) as well as balanced translocations involving *IGH* on Ch14q32, particularly t(4,14), are associated with a poor prognosis, while hyperdiploidy is associated with improved outcomes (Boyd *et al*, 2012; Sonneveld *et al*, 2016). Emergence of poor prognosis aberrations is postulated as one mechanism of resistant relapse; patients with multiple poor prognosis aberrations are described as having “ultra‐high‐risk” or “double‐hit” disease, and have an even higher risk of death and progression than those defined as “high‐risk” (one poor prognosis aberration) (Shah *et al*, 2018). However, there are few data available about the emergence of refractory clones in patients relapsing after autologous stem cell transplantation (ASCT).

The British Society of Bone Marrow Transplantation/UK Myeloma Forum (BSBMT/UKMF) Myeloma X Relapse (Intensive) trial (ISCRTN60123120), a study in first relapse of MM, aimed to evolve our understanding of the tumour genomic landscape by requiring chromosomal analysis for all patients at trial registration (baseline). Some participants also had chromosomal analysis results from diagnosis, the majority from the Medical Research Council (MRC) Myeloma IX trial (ISRCTN684564111), of which the cytogenetic procedures have been published previously (Ross *et al*, 2010).

The primary and secondary outcomes of the trial have been published previously (Cook *et al*, 2014, 2016). The analysis reported here had two aims. First, to update the previously reported time‐to‐event endpoints from randomisation, for which extended follow‐up data to a minimum of 5 years was available: Time to Progression (TTP), Progression‐Free Survival (PFS), second Progression‐Free Survival (PFS2) and Overall Survival (OS). Second, to investigate whether the presence of cytogenetic abnormalities at trial registration (baseline) were correlated with response, TTP, PFS, PFS2 and OS. Exploratory aims considered whether chromosomal analysis changed over time, reflecting sub‐clonal selection (between diagnosis and first relapse) and the effect of patients in the weekly cyclophosphamide arm receiving a subsequent salvage ASCT post‐trial (treatment switching) on OS.

## Methods

### Study design and patients

**Figure 1:**
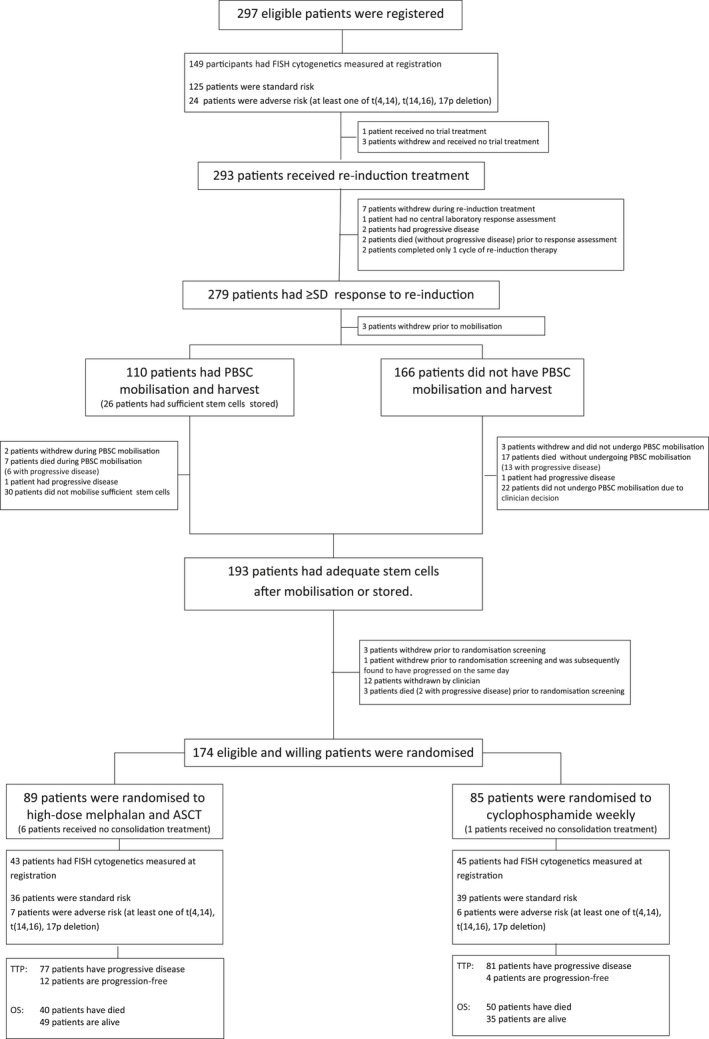
CONSORT Diagram for the BSBMT/UKMF Myeloma X relapse intensive trial. ASCT, autologous stem‐cell transplant; BSBMT, British Society of Bone Marrow Transplantation; FISH, fluorescence *in situ* hybridization; OS overall survival; PBSC, peripheral blood stem cells; SD, stable disease; TTP, time to progression; UKMF, UK Myeloma Forum.

The BSBMT/UKMF Myeloma X Relapse (Intensive) trial was a multicentre, randomised, open‐label, phase 3 trial. Patients with symptomatic, measurable MM were eligible for the trial if they required treatment for first progressive disease [as defined by the International Myeloma Working Group (IMWG) criteria (Durie *et al*, 2006)] at least 18 months following an ASCT (or 12 months following a trial amendment in 2011). Detailed inclusion and exclusion criteria have been published previously (Cook *et al*, 2014). The study was implemented at 51 National Health Service hospitals in the UK, for which written informed consent was obtained from all registered participants. The trial was approved by the Multi‐Centre Research Ethics Committee, UK, institutional review boards of the participating centres and by the competent regulatory authority (Medicines and Healthcare Products Regulatory Agency, UK). Additionally, the study was conducted according to the Declaration of Helsinki and the principles of International Conference on Harmonization Guidelines for Good Clinical Practice and was registered with an appropriate body (ISCRTN60123120). The trial procedures have been described previously (Cook *et al*, 2014), and are summarised in the trial CONSORT diagram (Fig 1) and in the Data S1, together with the randomisation details.

### Cytogenetic analyses

Plasma cells were isolated from fresh, unfixed bone marrow aspirate. Approximately 2 × 10^6^ marrow cells were incubated with CD138 antibody and plasma cells were recovered using an immunomagnetic cell selection process (Auto‐MACs, Miltenyi Biotec Ltd., Bisley, UK). The recovered plasma cells were fixed in a suspension in Carnoy's solution (three parts methanol and one part acetic acid) and stored at −20°C until iFISH testing.

iFISH was done in a two‐step manner using a threshold of 10%, with probes for 1p32.3 (*CDKN2C*), 1q21 (*CKS1B*), 13q14, *IGH* and *MYC* (all from Cytocell Ltd., Cambridge, UK) set up in a first round and, if an *IGH* rearrangement was identified, the additional tests for myeloma‐associated translocations *FGFR3/IGH* (t(4,14)), *MAF/IGH* (t(14,16)) and *CCND1/IGH* (t(11,14)) (all probes from Abbott Molecular, Maidenhead, UK) were done as a second round. Hyperdiploidy was inferred by the presence of an extra chromosome copy number.

An aliquot (2 μl) of fixed plasma cell suspension was placed on a marked area of a standard microscope slide for each test. The probes were prepared according to the manufacturer's guidance. The cells were air‐dried prior to probe application with no other pre‐treatment conditioning. Two microlitres of probe were added directly to the cell preparation and covered by a 12 mm diameter glass coverslip and sealed with rubber glue. The slides were placed on a programmable hot‐plate for co‐denaturation of probe and DNA/cells with time and temperature according to the manufacturer's protocol. Following overnight hybridisation at 37°C the slides underwent a stringency wash before counter‐staining with 4′,6‐diamidino‐2‐phenylindole (DAPI) for visualisation.

iFISH tests were assessed using an AxioPlan2 epi‐fluorescent microscope (Zeiss UK, Cambridge, UK) and images were captured using MetaSystems image capture software (https://metasystems-international.com/). All tests were checked by two scientists and results reported in line with the European Myeloma Network Guideline (Ross *et al*, 2012).

### Outcomes and definitions

For the extended follow‐up analysis TTP, OS, PFS and PFS2 were considered. The primary outcome, TTP, was defined as the time from randomisation to the first on‐trial record of progressive disease. OS was defined as the time from randomisation to death. PFS was defined as the time from randomisation to the first on‐trial record of progressive disease or death. Finally, PFS2 was defined as the time from randomisation to the second on‐trial record of progressive disease or death. If a participant had not experienced the event of interest by the time of the analysis, then they were censored at the time at which they were last known to be event‐free.

The subgroup analysis also considered response, defined as response following both re‐induction therapy and randomisation, using the IMWG criteria (Durie *et al*, 2006). The endpoint was categorised into; complete response [CR, also including stringent complete response (sCR)], partial response [PR, also including very good partial response (VGPR)], stable disease (SD), progressive disease (PD) and early death. For response following randomisation the endpoint was also categorised into ≥VGPR (sCR, CR or VGPR) and <VGPR (PR, SD, PD or early death).

The presence of the cytogenetic characteristics t(4;14), t(11;14), del(17p), 13q deletion [del(13q)], hyperdiploidy, *MYC* rearrangement and an individual's cytogenetic risk group at baseline [standard (no high‐risk lesions), adverse (any high‐risk lesion)] were investigated as subgroups. Note that t(4;14), t(14;16) and del(17p) were defined as high‐risk lesions. Other cytogenetic characteristics were considered for descriptive purposes only [t(14;16), *MYC* (Normal, re‐arranged, copy‐number)], and trichotomous risk groups [Standard (no high‐risk lesions), high‐risk (one high‐risk lesion), ultra‐high‐risk (more than one high‐risk lesion)].

The cytogenetic characteristics at baseline represent the results from the sample taken at trial registration, whereas the cytogenetic characteristics at diagnosis represent results from the original diagnosis (collected retrospectively in Myeloma X or prospectively if the participant was in MRC Myeloma IX). In the event that the cytogenetic characteristics of an individual had changed between original diagnosis and baseline the individual was categorised using the results at baseline for the subgroup analysis.

### Statistical analysis

Details of the sample size, recruitment and trial closure have been published previously (Cook *et al*, 2014, 2016). The cut‐off date for this analysis was 20 January 2017, when the 5‐year follow‐up was complete. Response has the cut‐off of 9‐July 2013. All analysis was in accordance with the Myeloma X (Relapse) Intensive Protocol (Version 7 September 2011 URL:https://ars.els-cdn.com/content/image/1-s2.0-S2352302616300497-mmc1.pdf), and was pre‐specified in the Myeloma X long‐term follow‐up Statistical Analysis Plan (dated 2015) and conducted using SAS 9.4 (SAS Institute, Cary, NC, USA), unless described as post‐hoc exploratory analysis, which was not pre‐specified and conducted using STATA 13 (StataCorp, College Station, TX, USA).

The intention‐to‐treat (ITT) analysis of TTP, PFS, OS, PFS2 and response following randomisation included all individuals randomised to the study. The various subgroup analysis included all randomised individuals with cytogenetic analysis for the subgroup under consideration, defined using the baseline sample. The cytogenetic subgroups were not mutually exclusive, *i.e*. patients may have multiple abnormalities detected. Response post‐induction included all registered individuals with cytogenetic analysis. Any changes of cytogenetic abnormalities between diagnosis and first relapse (baseline) were considered descriptively.

Cox proportional hazards regression was used to analyse the time‐to‐event endpoints, adjusting for the stratification factors (length of first remission or plateau and response to re‐induction treatment), and whether or not mobilisation treatment was received. These models were then extended to include appropriate interaction terms in order to assess whether the effect of the randomisation allocation on the time‐to‐event endpoints was heterogeneous between the levels of the investigated subgroups. Likelihood ratio tests (LRT) were used to test for treatment heterogeneity by subgroup. Fisher's exact tests were used to assess whether an association existed between response and the various cytogenetic subgroups. Response post‐randomization was analysed by logistic regression with the appropriate interaction term, adjusting for the stratification factors of the trial and whether or not peripheral blood stem cells (PBSC) mobilisation therapy was received, and an LRT conducted to test for treatment heterogeneity.

In an exploratory post‐hoc analysis, OS was considered using the rank‐preserving structural failure time model (RPSFTM) approach (White *et al*, 1999; Watkins *et al*, 2013) to take into account patients in the weekly cyclophosphamide group, who received a subsequent salvage ASCT for the treatment of second, third or subsequent relapse off‐trial. The RPSFTM method estimates the counterfactual randomised treatment effect by considering what the follow‐up time of the participant would have been if they had not switched to the alternative treatment, under the assumption that the overall treatment effect is constant irrespective of when the treatment switch occurs.

This study is registered with ClinicalTrials.gov (NCT00747877) and EudraCT (2006‐005890‐24).

## Results

Two hundred and ninety‐seven patients were registered to the trial between 16 April 2008 and 19 November 2012, of which 149 patients (50·2%) had cytogenetic analysis at baseline. The ITT population considered 174 randomised participants (Table 1), 88 (50·5%) of which had cytogenetic analysis at baseline, 43 (48·9%) in the salvage ASCT group and 45 (51·1%) in the weekly cyclophosphamide group. There were no substantial differences between patients with and without cytogenetic analysis at baseline (Table SI). Similarly, there were no substantial differences in the baseline characteristics of the randomised groups when considering those who had cytogenetic analysis measured at; diagnosis, first relapse (baseline) and both, or at least one time point (Tables SII–SV).

**Table 1:** Baseline characteristics of patients in the intention‐to‐treat population

	Registered (*n* = 297)	Salvage ASCT (*n* = 89)	Weekly cyclophosphamide (*n* = 85)	Randomised (*n* = 174)
Age at baseline, years				
Median (IQR)	61 (55, 65)	61 (56, 64)	61 (54, 65)	61 (56, 65)
Patient gender				
Male	208 (70·0%)	65 (73·0%)	61 (71·8%)	126 (72·4%)
Female	89 (30·0%)	24 (27·0%)	24 (28·2%)	48 (27·6%)
Patient race				
White	267 (89·9%)	81 (91·0%)	80 (94·1%)	161 (92·5%)
Mixed – White and Black Caribbean	1 (0·3%)	0 (0·0%)	0 (0·0%)	0 (0·0%)
Asian – Indian	3 (1·0%)	1 (1·1%)	2 (2·4%)	3 (1·7%)
Asian – Pakistani	1 (0·3%)	1 (1·1%)	0 (0·0%)	1 (0·6%)
Asian – Bangladeshi	1 (0·3%)	0 (0·0%)	0 (0·0%)	0 (0·0%)
Other Asian background	2 (0·7%)	1 (1·1%)	0 (0·0%)	1 (0·6%)
Black – Caribbean	8 (2·7%)	2 (2·2%)	1 (1·2%)	3 (1·7%)
Black – African	4 (1·3%)	1 (1·1%)	0 (0·0%)	1 (0·6%)
Other Black background	2 (0·7%)	0 (0·0%)	1 (1·2%)	1 (0·6%)
Other ethnic group	2 (0·7%)	0 (0·0%)	0 (0·0%)	0 (0·0%)
Not stated	3 (1·0%)	2 (2·2%)	1 (1·2%)	3 (1·7%)
Missing	3 (1·0%)	0 (0·0%)	0 (0·0%)	0 (0·0%)
Paraprotein type				
IgG	190 (64·0%)	60 (67·4%)	57 (67·1%)	117 (67·2%)
IgA	55 (18·5%)	13 (14·6%)	18 (21·2%)	31 (17·8%)
IgM	1 (0·3%)	1 (1·1%)	0 (0·0%)	1 (0·6%)
IgD	2 (0·7%)	0 (0·0%)	1 (1·2%)	1 (0·6%)
Light chain only	28 (9·4%)	7 (7·9%)	7 (8·2%)	14 (8·0%)
Non‐secretor	9 (3·0%)	3 (3·4%)	2 (2·4%)	5 (2·9%)
Missing	12 (4·0%)	5 (5·6%)	0 (0·0%)	5 (2·9%)
Light chain type				
Lambda	82 (27·6%)	24 (27·0%)	21 (24·7%)	45 (25·9%)
Kappa	185 (62·3%)	52 (58·4%)	59 (69·4%)	111 (63·8%)
Missing	30 (10·1%)	13 (14·6%)	5 (5·9%)	18 (10·3%)
ISS at baseline				
I	188 (63·3%)	57 (64·0%)	51 (60·0%)	108 (62·1%)
II	61 (20·5%)	18 (20·2%)	21 (24·7%)	39 (22·4%)
III	27 (9·1%)	8 (9·0%)	4 (4·7%)	12 (6·9%)
Missing	21 (7·1%)	6 (6·7%)	9 (10·6%)	15 (8·6%)
Previous treatment response length				
<18 months	N/A	3 (3·4%)	2 (2·4%)	5 (2·9%)
18–24 months	N/A	22 (24·7%)	19 (22·4%)	41 (23·6%)
>24 months	N/A	64 (71·9%)	64 (75·3%)	128 (73·6%)
Response to re‐induction treatment				
SD	N/A	7 (7·9%)	5 (5·9%)	12 (6·9%)
More than PR (PR, VGPR, CR or sCR)	N/A	82 (92·1%)	80 (94·1%)	162 (93·1%)
PBSC mobilisation and harvest given				
Yes	N/A	44 (49·4%)	26 (30·6%)	70 (40·2%)
No	N/A	43 (48·3%)	56 (65·9%)	99 (56·9%)
Missing data	N/A	2 (2·2%)	3 (3·5%)	5 (2·9%)
iFISH cytogenetic results at diagnosis				
Yes	63 (21·2%)	20 (22·5%)	26 (30·6%)	46 (26·4%)
No	234 (78·8%)	69 (77·5%)	59 (69·4%)	128 (73·6%)
iFISH cytogenetic results at baseline				
Yes	149 (50·2%)	43 (48·3%)	45 (52·9%)	88 (50·6%)
No	148 (49·8%)	46 (51·7%)	40 (47·1%)	86 (49·4%)
iFISH cytogenetic results at diagnosis and baseline				
Yes	41 (13·8%)	13 (14·6%)	17 (20·0%)	30 (17·2%)
No	256 (86·2%)	76 (85·4%)	68 (80·0%)	144 (82·8%)
iFISH cytogenetic results at diagnosis or baseline				
Yes	171 (57·6%)	50 (56·2%)	54 (63·5%)	104 (59·8%)
No	126 (42·4%)	39 (43·8%)	31 (36·5%)	70 (40·2%)

ASCT, autologous stem‐cell transplant; CR, complete response; iFISH, interphase fluorescence *in situ* hybridization; IQR, inter‐quartile range; ISS, International Staging System; PBSC, peripheral blood stem cells; PR, partial response; sCR, stringent complete response; SD, stable disease; VGPR, very good partial response.

**Figure 2:**
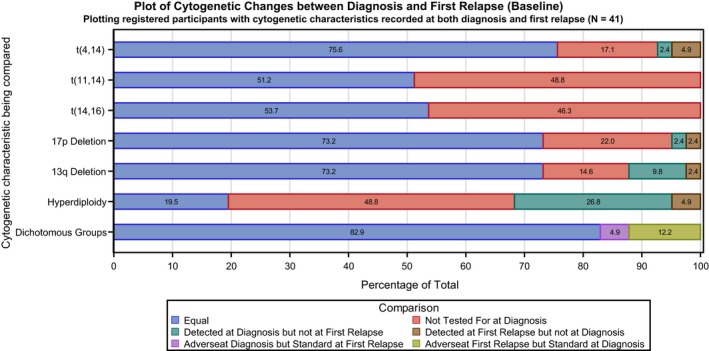
Comparison of the fluorescence *in situ* hybridization cytogenetic analysis for registered individuals who had cytogenetic results at diagnosis and first relapse (baseline).

Forty‐one registered patients had cytogenetic results at diagnosis and first relapse (baseline). The majority of patients [*n* = 34 (82·9%)] had the same cytogenetic risk group at both time points (Fig 2). The largest variation within the individual cytogenetic components was in hyperdiploidy; 11 (26·8%) patients showed hyperdiploidy at diagnosis but not at first relapse while two (4·9%) patients gained this feature. Similarly, four (9·8%) patients had del(13q) at diagnosis but not at first relapse, with one (2·4%) patient gaining this characteristic at first relapse. In summary, five (12·2%) patients evolved to high‐risk disease at first relapse, having been defined as standard risk at diagnosis, while two (4·9%) patients became standard risk at first relapse despite being high‐risk at diagnosis with four (9·8%) patients retaining high‐risk cytogenetics at both times. Note that *MYC* was not considered here as it was not tested for at diagnosis.

As has previously been demonstrated in first‐line therapy, there was no evidence to suggest an association between any of the genetic abnormalities studied and depth of response (sCR or CR VGPR or PR, SD or PD) following re‐induction or randomisation to consolidation. Similarly, no impact on depth of response (<VGPR or ≥VGPR) was evident post‐randomisation (Figure S1).

**Figure 3:**
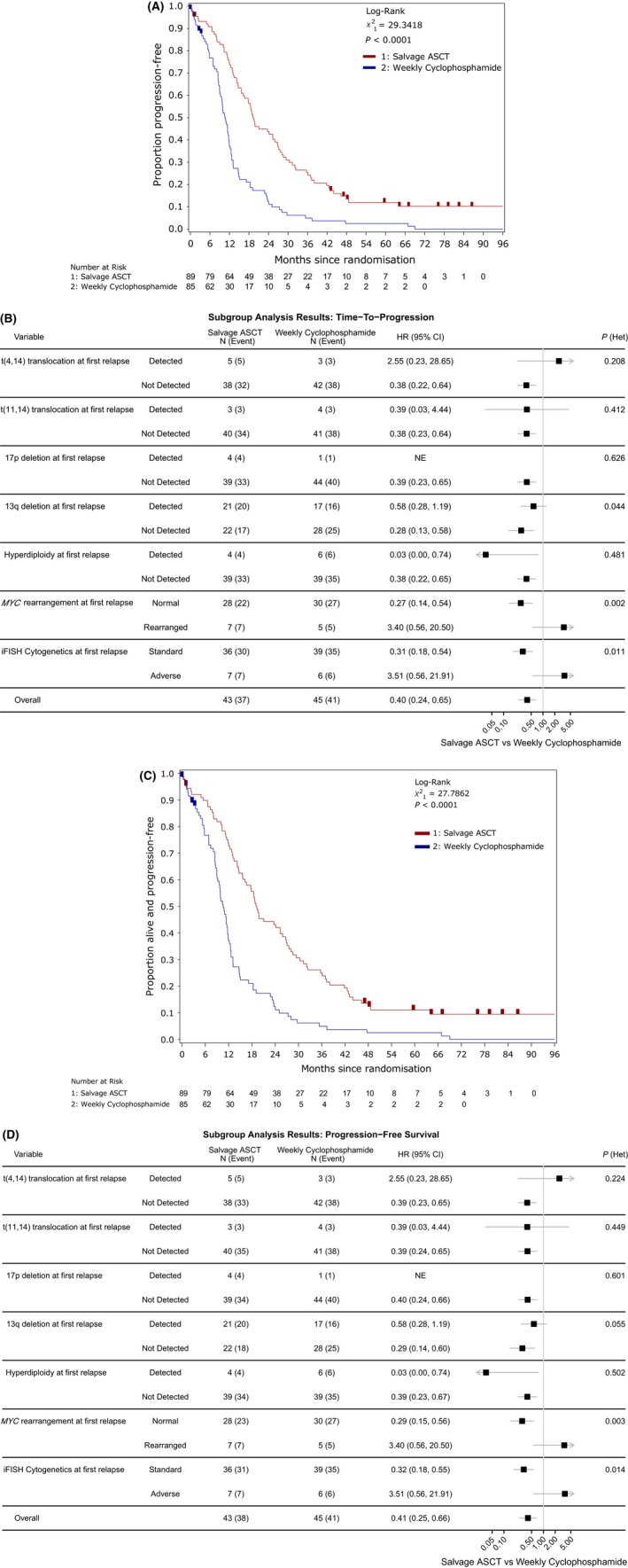
TTP and PFS analysis results. (A) TTP by randomised treatment. (B) Forest plot of the subgroup analysis for TTP. (C) PFS by randomised treatment. (D) Forest plot of the subgroup analysis for PFS. The black squares and horizontal lines represent the hazard ratio (HR) and the associated 95% confidence interval (95% CI) of the risk of progression (TTP) and progression or death (PFS) in the Salvage ASCT treatment arm compared to the weekly cyclophosphamide arm, p(het) represents the *P*‐value from the likelihood ratio test assessing heterogeneity of treatment effect between subgroups. ASCT, autologous stem‐cell transplant; CI, confidence interval; HR, hazard ratio; iFISH, interphase fluorescence *in situ* hybridization; NE, not evaluable; PFS, progression‐free survival; TTP, time to progression.

The median follow‐up of this study was 76 months (Inter‐quartile range (IQR): 60–83 months). In total there have been 158 disease progressions of 174 randomised patients *i.e*. 90·8% of all randomized participants have had documented PD on trial. In the salvage ASCT group there have been 77 (86·5%) progressions compared to 81 (95·3%) in the weekly cyclophosphamide group. The updated TTP analysis continues to show a significant advantage for salvage ASCT compared to weekly cyclophosphamide [19 months (95% confidence interval (95% CI): 16–26 vs. 11 months (9–12)]. Figure 3A shows the Kaplan‐Meier curve for TTP stratified by randomisation allocation. Cox proportional hazards regression (adjusted for stratification factors and PBSC mobilisation) showed a reduced hazard of progression in the salvage ASCT group compared with weekly cyclophosphamide [hazard ratio (HR): 0·40, 95% CI: 0·29–0·56, *P* < 0·001].

The treatment effects for those with cytogenetic analysis at baseline and the whole ITT population are consistent (HR: 0·40, 95% CI: 0·24–0·65). Figure 3B shows a forest plot of the TTP results correlated with the cytogenetic analysis at baseline. There is significant evidence of heterogeneity of treatment effect with del(13q) (LRT: *P* = 0·044) and *MYC* rearrangements (LRT: *P* = 0·002). This heterogeneity of treatment effect is also observed in patients with standard and adverse cytogenetics (LRT: *P* = 0·011). In each case the presence of the marker has a deleterious impact on the randomised treatment effect observed, compared with the subset of patients without the marker. The deleterious impact of the presence of *MYC* rearrangements can be seen further in Kaplan‐Meier curves for TTP, stratified by whether *MYC* was rearranged or normal for each treatment allocation (Figure S2).

There have been 160 PFS events in 174 randomised patients (92·0%). In the salvage ASCT group, 79 PFS events (88·8%) have been confirmed compared to 81 (95·3%) in the weekly cyclophosphamide group. Median PFS was 19 months (95% CI: 16–25) for the salvage ASCT group and 11 months (9–12) for the weekly cyclophosphamide group. Figure 3C shows the Kaplan‐Meier curve for PFS stratified by randomisation allocation. Cox proportional hazards regression (adjusted for stratification factors and PBSC mobilisation) showed a reduced hazard of death in the salvage ASCT group compared with weekly cyclophosphamide (HR: 0·41, 95% CI: 0·30–0·58, *P* < 0·001).

The treatment effects for those with cytogenetic analysis at baseline and the ITT population are consistent (HR: 0·41, 95% CI: 0·25–0·66). Figure 3D shows a forest plot of the PFS results correlated with the cytogenetic analysis at baseline. There is significant evidence of heterogeneity of treatment effect with *MYC* rearrangement (LRT: *P* = 0·003) as well as standard *versus* adverse comparisons (LRT: *P* = 0·014). In each case the presence of the marker has a deleterious impact on the randomised treatment effect observed, compared with the subset of patients without the marker.

**Figure 4:**
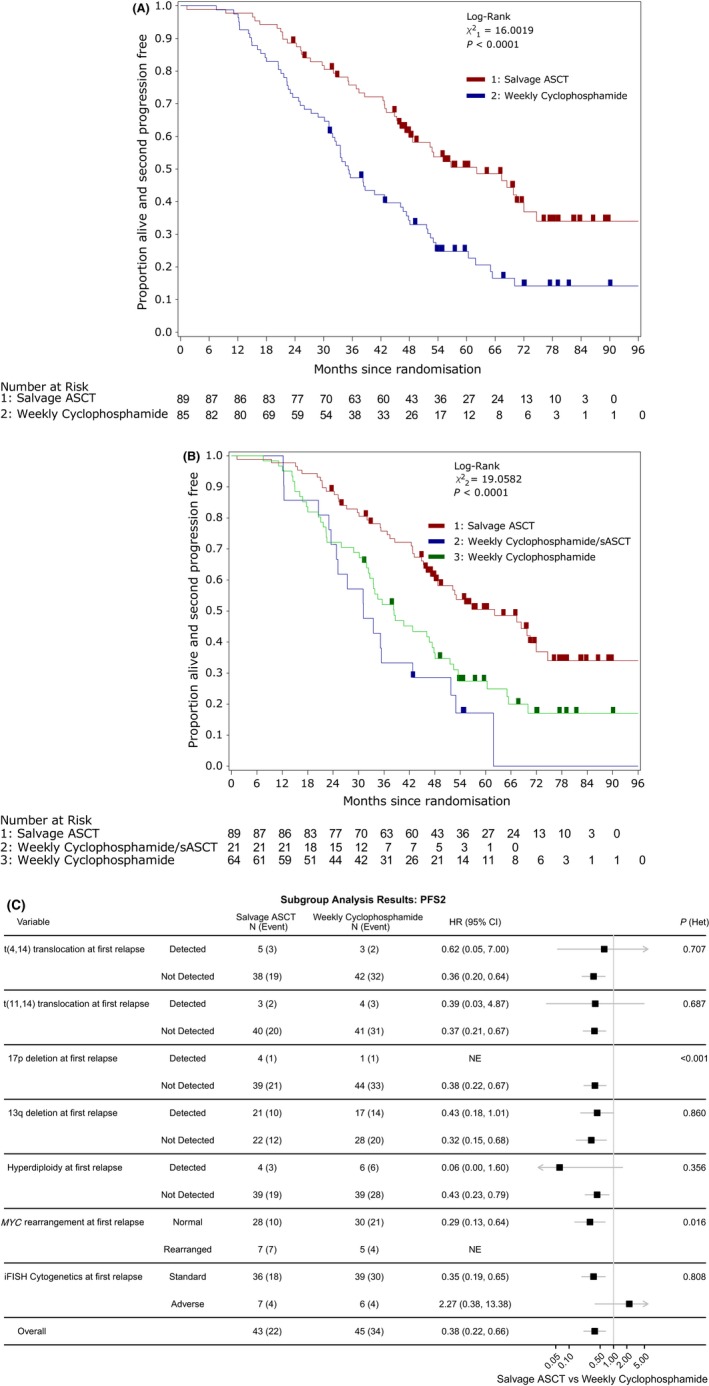
PFS2 analysis results. (A) By randomised treatment. (B) By randomised treatment with the weekly cyclophosphamide group separated by those who receive a subsequent ASCT (sASCT). (C) Forest plot of the subgroup analysis conducted for PFS2. The black squares and horizontal lines represent the hazard ratio (HR) and the associated 95% confidence interval (95% CI) of the risk of second progression or death in the Salvage ASCT treatment arm compared to the weekly cyclophosphamide arm, p(het) represents the *P*‐value from the likelihood ratio test assessing heterogeneity of treatment effect between subgroups. ASCT, autologous stem‐cell transplant; CI, confidence interval; HR, hazard ratio; iFISH, interphase fluorescence *in situ* hybridization; PFS2; second progression‐free survival; sASCT, subsequent autologous stem‐cell transplant.

There have been 112 PFS2 events in 174 randomised patients (64·4%), with 47 second progressions or deaths (52·8%) confirmed in the salvage ASCT group compared to 65 (76·5%) in the weekly cyclophosphamide group. Median PFS2 was 62 months (95% CI: 47–72) for the salvage ASCT group and 35 months (31–46) in the weekly cyclophosphamide group. The adjusted Cox proportional hazard regression showed a reduced hazard of second progression or death in the salvage ASCT group compared with weekly cyclophosphamide (HR: 0·45, 95% CI 0·30–0·66, *P* < 0·001). Figure 4A shows the Kaplan‐Meier curve for PFS2 stratified by randomisation allocation. Figure 4B extends this by considering those in the weekly cyclophosphamide group who had a salvage ASCT post‐trial and those who did not (median PFS2 31 months (95% CI: 24–43) vs. 38 months (32–48) respectively).

Figure 4C shows a forest plot of the PFS2 results correlated with the cytogenetic analysis at baseline. The treatment effects for those with cytogenetic analysis at baseline and the ITT population are consistent (HR: 0·38, 95% CI: 0·22–0·66). Whilst an HR is not estimable for the rearranged subgroup, there is significant evidence of heterogeneity of treatment effect with *MYC* rearrangement (LRT: *P* = 0·016).

**Figure 5:**
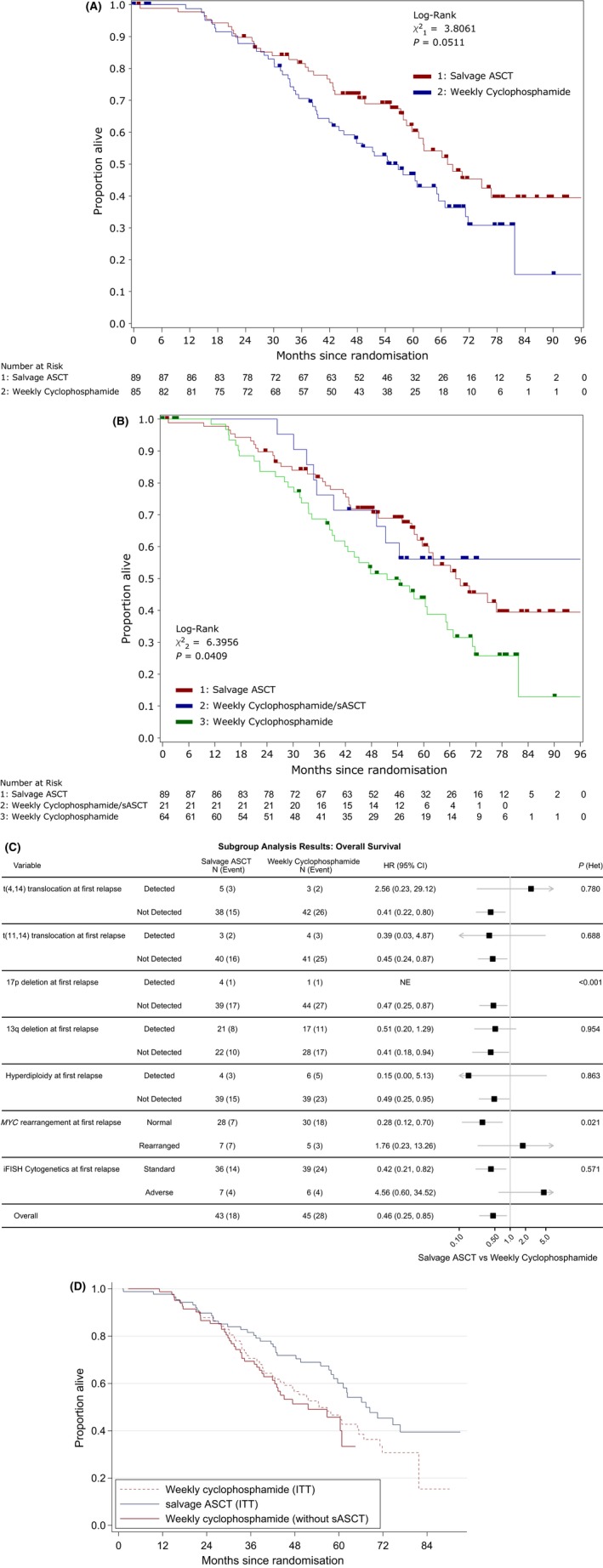
Overall survival analysis results. (A) By randomised treatment. (B) By randomised treatment with the weekly cyclophosphamide group separated by those who receive a subsequent ASCT (sASCT). (C) Forest plot of the subgroup analysis conducted for OS. The black squares and horizontal lines represent the hazard ratio (HR) and the associated 95% confidence interval (95% CI) of the risk of death in the Salvage ASCT treatment arm compared to the weekly cyclophosphamide arm, p(het) represents the *P*‐value from the likelihood ratio test assessing heterogeneity of treatment effect between subgroups. (D) By randomised treatment using the rank‐preserving structural failure time model (RPSFTM) to account for treatment switching from weekly cyclophosphamide to salvage ASCT. ASCT, autologous stem‐cell transplant; CI, confidence interval; HR, hazard ratio; iFISH, interphase fluorescence *in situ* hybridization; ITT, intention to treat; OS, overall survival; sASCT, subsequent autologous stem‐cell transplant.

The extended median OS follow‐up is 70 months (IQR: 55–79 months) with 125 (42·1%) registered trial patients having died. Of these, 90 patients have died following randomisation, *i.e*. 51·7% of randomized participants have died. In the salvage ASCT group there have been 40 (44·9%) deaths compared to 50 (58·8%) in the weekly cyclophosphamide group. Disease progression was responsible for 63·3% (salvage ASCT: 60·0%, weekly cyclophosphamide: 66·0%) of deaths. Median OS was 67 months [95% CI: 59‐not reached (NR)] for the salvage ASCT group and 55 months (44–67) for the weekly cyclophosphamide group. Adjusted Cox proportional hazards regression showed a reduced hazard of death in the salvage ASCT group compared with weekly cyclophosphamide (HR: 0·64, 95% CI: 0·42–0·99, *P* = 0·0435); a similar result to our previous shorter‐term results (Cook *et al*, 2016). Figure 5A shows the Kaplan‐Meier curve for OS stratified by randomisation allocation. Figure 5B extends this by considering those in the weekly cyclophosphamide group who had a subsequent salvage ASCT following the trial and those who did not [median OS NR months (95% CI: 39‐NR) vs. 31 months (22–39), respectively].

Figure 5C shows a forest plot of the OS results correlated with cytogenetic analysis at baseline. The treatment effects for those with cytogenetic analysis at baseline and the ITT population are consistent (HR: 0·46, 95% CI: 0·25–0·85). There is significant evidence of heterogeneity of treatment effect with *MYC* rearrangement (LRT: *P* = 0·021), in which the presence of the marker has a deleterious impact on the randomized treatment effect observed, compared with the subset of patients without the marker. This deleterious impact can be seen further in a Kaplan‐Meier curve for OS stratified by whether *MYC* was rearranged or normal for each treatment allocation (Figure S3). Whilst del(17p) is shown to have significant evidence to heterogeneity (LRT: *P* < 0·001) an exact HR cannot be calculated for the detected subgroup as only one participant had the marker in the weekly cyclophosphamide group.

This long‐term follow‐up analysis has permitted use of a technique developed for the analysis of drug registration trials, in which the investigational drug is permitted post‐trial in the control comparator group. In this situation this relates to the subsequent delivery in later line therapy of salvage transplant in patients allocated to the weekly cyclophosphamide group. In the weekly cyclophosphamide group 16 patients (24·2%) received a subsequent salvage ASCT as part of their third‐line of therapy, four patients (8·5%) as part of their fourth‐line of therapy and one patient (6·3%) as part of a later‐line of therapy. These subsequent salvage ASCTs have diluted the allocated treatment effect in terms of OS, rescuing some patients after their non‐transplant consolidation on trial at first relapse. This can be seen in Fig 5D where there is a difference between the ITT weekly cyclophosphamide estimate of OS and the counterfactual estimate of OS for that group accounting for the subsequent salvage ASCT. The counterfactual estimate of median OS for the weekly cyclophosphamide group was 52 months (95% CI: 41‐NR). This reflects the impact of a subsequent salvage ASCT in 21 of 85 patients (24·7%) in the weekly cyclophosphamide group, leading to an improvement in median OS of 3 months in the ITT estimate of OS. The counterfactual HR for the adjusted analysis is smaller than that for the ITT analysis (ITT HR: 0·63, 95% CI: 0·42–0·96, *P* = 0·033; adjusted HR: 0·52, 95% CI: 0·29–0·95), similarly indicating the rescue effect of a subsequent salvage ASCT. There was no evidence of the assumption of constant treatment effect being violated in the counterfactual analysis (Figure S4), as the 95% CI for the estimated survivor functions by group, where the effect of salvage ASCT was removed, overlapped. This could suggest that the effect of a subsequent salvage ASCT is, in fact, similar to that delivered at first relapse, conditional on a patient surviving to that subsequent line of treatment. Another possible reason for the limited impact of later second transplant on these patients is the number of non‐transplanted patients (~79%) who received second generation immunomodulatory drugs (IMiDs) post‐trial. Details of the therapies given post‐trial are shown in Table 2. Excluded from Table 2 are the small number of patients who received steroids with or without alkylating agents, local radiotherapy and other agents used on only one patient per cohort.

**Table 2:** Details of treatment following second, third and subsequent relapse^a^

	Second relapse		Third relapse		Subsequent relapse	
	Salvage ASCT (*N* = 69)	Weekly Cyclophosphamide (*N* = 66)	Salvage ASCT (*N* = 26)	Weekly cyclophosphamide (*N* = 47)	Salvage ASCT (*N* = 10)	Weekly cyclophosphamide (*N* = 16)
Subsequent ASCT	0 (0%)	16 (24·2%)	0 (0%)	4 (8·5%)	0 (0%)	1 (6·3%)
Allo SCT	1 (1·4%)	1 (1·5%)	0 (0%)	0 (0%)	0 (0%)	0 (0%)
Thalidomide	7 (10·1%)	3 (4·5%)	7 (26·9%)	4 (8·5%)	3 (30%)	7 (43·8%)
Bortezomib	5 (7·2%)	14 (21·2%)	8 (30·8%)	12 (25·5%)	0 (0%)	3 (18·8%)
Lenalidomide	60 (87·0%)	52 (78·8%)	5 (19·2%)	19 (40·4%)	1 (10%)	2 (12·5%)
Pomalidomide	2 (2·9%)	0 (0%)	5 (19·2%)	15 (31·9%)	4 (40%)	11 (68·8%)
Others	4 (5·8%)	0 (0%)	1 (3·8%)	4 (8·5%)	1 (10%)	3 (18·8%)

Allo SCT, allogeneic haematopoietic stem cell transplantation; ASCT, autologous stem cell transplant.

a Treatments are non‐mutually exclusive.

## Discussion

Depth of response to therapy in MM, especially to below the level of minimal residual disease detection, is associated with durability of response and survival in first‐line therapy (Lonial & Anderson, 2014; Munshi *et al*, 2017). However, several features can influence this durability, none more significantly than the genomic landscape at presentation of disease activity requiring therapy. Much of this evidence has been generated in the frontline setting with an accumulation of data demonstrating the significance in relapsed disease management (Hébraud *et al*, 2013). In Myeloma X, the first randomised study of salvage ASCT at first relapse, we have already shown that depth of response (in relation to TTP and PFS) to a salvage ASCT equates to a survival advantage (Cook *et al*, 2016), and in this report we suggest that genomic adverse markers at relapse also influence the outcome of second‐line therapy. This report highlights a number of key issues.

The current study provides the first evidence of changes in the clonal genetic landscape in the setting of ASCT and salvage ASCT. Several publications have highlighted the sub‐clonal nature of MM, and how alternative clones become dominant at relapse, a feature which may ultimately provide a rational for changing treatment strategies (Sonneveld *et al*, 2016). Nonetheless, this evidence is somewhat limited and anecdotal. We have provided linked serial evidence that highlights clonal heterogeneity and evolution, in the setting of a clinical interventional study with access to pre‐trial biological information. Our evidence shows that whilst many patients, following first relapse after a prior ASCT, retain their cytogenetic findings from diagnosis, some patients develop adverse cytogenetic characteristics while others lose theirs (or they are at least reduced to undetectable levels) – a feature that to date has not been reported in MM. This highlights sub‐clonal selection through the use of novel agents and prior ASCT. The longitudinal study data presented here helps to confirm the findings of the Intergroupe Francophone du Myélome (Hébraud *et al*, 2013), but note these were not observed by the longitudinal German‐speaking Myeloma Multicentre Group (GMMG) study, where acquisition of additional (adverse) cytogenetic findings (seen in approximately one in eight of our patients) was well recognised, but where loss of poor prognostic findings (seen in one in 20 of our patients) was not frequently described (Merz *et al*, 2017). Unfortunately, data on *MYC* rearrangements were not available for patients at diagnosis and evidence for inclusion of 1q21 gain was lacking when Myeloma X was designed, both of which would further the relevance of our findings. It has to be borne in mind that the trial population represents a selective group, given that the entry criteria stipulated a minimum of 18 months from ASCT to trial entry (or 12 months if after the trial amendment), thus potentially excluding those with high‐risk genetic disease. Therefore, it is somewhat surprising that 16·1% of all registered patients with baseline cytogenetic data (24 of 149) harboured high‐risk genetic aberrations, equating to 14·8% (13 of 88) of those randomised. However, it must be noted that only half of all registered participants (50·2%) had cytogenetic analysis recorded at baseline, equating to a similar proportion within those randomised.

The primary aim of any genetic analysis in a MM trial is to highlight prognostic groups (Palumbo *et al*, 2015), such that one can define if the experimental arm of a study can fully restore any durability of response and survival benefit associated with that intervention (“level the playing field”). In this analysis, whilst the majority of high‐risk markers [del(17p), t(4;14) and t(14;16)], when considered individually, do not prevent the PFS and OS advantage of a salvage ASCT compared to weekly cyclophosphamide, the negative influence of the poor prognostic marker, *MYC* rearrangement, prevails. The level of *MYC* rearrangements in the study populations of key relapse phase 3 studies is not usually documented (Lonial *et al*, 2015; Stewart *et al*, 2015; Dimopoulos *et al*, 2016; Moreau *et al*, 2016), so defining its universality of impact becomes difficult. *MYC* activation, either through translocation or gain of the *MYC* locus, results in deregulation of upstream pathways such as IRF4 or MAPK (Jovanović *et al*, 2018). A better understanding of the mechanisms responsible for *MYC* deregulation in MM offers the potential for more targeted approaches, given that high dose alkylating agent therapy used in salvage ASCT has shown limited effect on the adverse prognostic impact of *MYC* rearrangements. Several such therapeutic strategies for patients with MM are currently being evaluated in clinical trials, including Bromodomain and Extra‐Terminal (BET) inhibitors (Jovanović *et al*, 2018).

In analysing our data, the application of the RPSFTM methodology, as a post‐hoc exploratory analysis, to the detailed follow‐up of patients entered in this trial has produced evidence that ASCT, even if delayed to second or possibly third relapse, may be of benefit for patients having response to first ASCT of greater than 12 months. This is highly relevant to clinical decision‐making in this setting. As the trial met its primary endpoint early, only 174 eligible patients were randomised compared to the original estimate of 320; this resulted in an approximately 45% reduction in the sample size for the associated scientific studies. Whilst the baseline characteristics of those with and without cytogenetic analysis are similar (Table SI), the subgroup analysis results should still be interpreted with caution as hypothesis‐generating. However, the findings may be important for the design of future studies which may compare second ASCT to current therapies. This is particularly relevant in this study where the median OS of the control group has lengthened (but remains significantly shorter than the experimental arm) with prolonged follow‐up and is thought to be as a consequence of more recent novel therapeutic agent development and incorporation into the standard of care (SoC), such as second generation IMiDs and proteasome inhibitors. This too could explain the observed difference between PFS2 and OS, with the maturity of follow‐up presented in this last analysis. Measuring OS in clinical interventional studies, whilst of great importance, has become more difficult given the plethora of novel agents accessible in both SoC and through clinical interventional studies. In essence, not only are there treatment switch effects, from participants in the control arm accessing the study's “experimental treatment” following the completion of their trial treatment, i.e. salvage ASCT in the third‐line or later, but also clear evidence of post‐trial selection bias, in that patients with better performance status and stable haematopoietic reserve are able to access clinical trials and thus be exposed to newer efficacious agents in the phase 2 or even phase 3 setting, where as those who are not as fit or as biologically robust, may not. As such, the post‐trial therapeutic landscape provides significant heterogeneity and can diminish the survival impact from the primary trial intervention. However, each of these findings are beyond the main randomised question of the study and should be validated.

In summary, we have shown that the gains in TTP, PFS and OS observed from Myeloma X are robust, and confirmed at later follow‐up. This remains the only randomised evidence for salvage ASCT ahead of the reporting of GMMG ReLApsE trial (ISRCTN16345835). The ReLApsE study has an immunomodulatory agent control group, which is widely considered to be superior to weekly cyclophosphamide, and should provide further evidence for or against salvage ASCT with contemporary therapies. The genomic landscape with relapsing disease can vary from that seen at diagnosis with both loss and gain of adverse iFISH prognostic factors and confirm that the gain of such factors does affect prognosis in patients at first relapse. In particular, we highlight the adverse impact of *MYC* re‐arrangements, and that the current clinical interventions do not circumvent this adversity, highlighting the need for newer targeted strategies for this sub‐group of patients. Additionally, we present evidence suggesting that a second, salvage transplant can be of benefit for OS, even if delivered later than first relapse.

## Disclosures and Competing Interests

GC: Takeda – consultancy, honoraria, research funding, speakers bureau; Glycomimetics – consultancy, honoraria; Sanofi – consultancy, honoraria, speakers bureau; Celgene Corporation – consultancy, honoraria, research funding, speakers bureau; Janssen – consultancy, honoraria, research funding, speakers bureau; Bristol‐Myers Squibb – consultancy, honoraria; Amgen – consultancy, honoraria, research funding, speakers bureau. K‐LR: Celgene Corporation, Amgen, Merck Sharp and Dohme, Takeda – research funding. SO'C: None. DAC: Celgene Corporation, Amgen, Merck Sharp and Dohme, Takeda – research funding. AJA: Amgen – consultancy, honoraria and research funding; Celgene Corporation – consultancy, honoraria and research funding; Janssen – consultancy and research funding, Takeda – Consultancy and honoraria. CDW: Takeda – honoraria, travel support, speakers bureau; Amgen – honoraria, speakers bureau; Novartis – honoraria; Janssen – honoraria, travel support, speakers bureau; Celgene Corporation – honoraria, travel support, speakers bureau. AH: Celgene Corporation, Amgen, Merck Sharp and Dohme, Takeda – research funding. JDC: Celgene Corporation research funding, speakers bureau; Janssen – research funding, speakers bureau. JAS: Merck Sharp and Dohme – consultancy and educational support, speakers bureau; Janssen – consultancy and educational support, speakers bureau; Celgene Corporation – consultancy and educational support, speakers bureau; Sanofi – consultancy and educational support, speakers bureau. DA: Takeda – honoraria, travel support. ET: Jazz, Janssen, Amgen – speaker fees; Jazz, Pfizer, Amgen, MSD, Gilead – honoraria. VEA: None. MJ: Janssen – consultancy, honoraria, travel support, research funding; Takeda – consultancy, honoraria, travel support; Amgen – consultancy, honoraria, travel support; Celgene Corporation – consultancy, honoraria, research funding; Novartis – consultancy, honoraria. CP: Janssen and Celgene Corporation – speakers bureau. KY: Amgen – consultancy, honoraria; Novartis – consultancy, honoraria; Takeda – consultancy, honoraria; Bristol‐Myers Squibb – consultancy, honoraria; Janssen – consultancy, honoraria. JC: Celgene – speakers bureau, research funding Janssen – speakers bureau, research funding. HH: Celgene Corporation, Jazz – travel support; Jazz, Novartis – speaker fees. JMBi: Janssen – educational support, consultancy, speakers bureau, research funding; Roche – research funding; Celgene Corporation – educational support, consultancy, speakers bureau, research funding; Amgen – educational support, consultancy, speakers bureau; Pfizer – educational support, consultancy, speakers bureau; Bayer – research funding. GP: Janssen, Celgene Corporation, Takeda; Amgen; Gilead; Binding Site Ltd – medical advisory board. MTD: Abingdon Health – equity ownership, membership on an entity's board of directors or advisory committees. JMBr: Celgene Corporation, Amgen, Merck Sharp and Dohme, Takeda – research funding. TCMM: Janssen and Celgene Corporation – meeting support.

## Role of the funding source

The primary funder of the trial was Cancer Research UK (A7264), with financial contributions also received from Janssen‐Cilag and Chugai Pharma, UK. The funders had no role in study design, data collection, data analysis, data interpretation or writing of the report. The study was designed by GC, TCMM, JMBr and the Trial Management Group, on behalf of the UKMF and BSBMT. Data collection and the analysis were done by the Clinical Trials Research Unit, University of Leeds. The Trial Management Group, chaired by GC, verified the accuracy and completeness of the data reported and the adherence of the study to the protocol, in accordance with the principles of good clinical practice. DAC vouches for the statistical accuracy of the article. TCMM wrote the first draft of the article, and, along with the GC, made the decision to submit the article for publication in agreement with all the investigators participating in the trial. All authors had full access to the data and reviewed and approved the article before submission.

## Supporting information


**Data S1.** Supplementary Methods.
**Table SI.** Baseline demographics of patients with FISH cytogenetic analysis at trial registration (first relapse) and those not.
**Table SII.** Characteristics of those with cytogenetic data at diagnosis by randomisation allocation.
**Table SIII.** Characteristics of those with cytogenetic data at relapse by randomisation allocation.
**Table SIV.** Characteristics of those with cytogenetic data at diagnosis and relapse by randomisation allocation.
**Table SV.** Characteristics of those with cytogenetic data at diagnosis or relapse by randomisation allocation.
**Figure S1.** Forest plot of the complete cytogenetic subgroup results for Response. The black squares and horizontal lines represent the odds of a ≥ VGPR response in the salvage ASCT arm compared to the weekly cyclophosphamide arm and the associated 95% confidence interval, p(het) represents the p‐value from the likelihood ratio test assessing heterogeneity of treatment effect between subgroups.
**Figure S2.** Kaplan‐Meier curve for TTP by whether MYC was normal or rearranged at first relapse in patients randomised to (a) salvage ASCT and (b) weekly cyclophosphamide.
**Figure S3.** Kaplan‐Meier curve for OS by whether MYC was normal or rearranged at first relapse in patients randomised to (a) salvage ASCT and (b) weekly cyclophosphamide.
**Figure S4.** Kaplan‐Meier curve for OS with estimated 95% confidence intervals by randomised treatment assuming no treatment effect in either arm i.e. assuming neither group received an ASCT at any time point.
