# Supplementary material for: The impact of cytogenetics on duration of response and overall survival in patients with relapsed multiple myeloma (long‐term follow‐up results from BSBMT/UKMF Myeloma X Relapse [Intensive]): a randomised, open‐label, phase 3 trial
Source: Br J Haematol. 2019 Feb 6;185(3):450–67. doi: 10.1111/bjh.15782 (PMC6519200; doi:10.1111/bjh.15782)
Supplement: Supplementary file 1 — Data S1. Supplementary Methods. Table SI. Baseline demographics of patients with FISH cytogenetic analysis at trial registration (first relapse) and those not. Table SII. Characteristics of those with cytogenetic data at diagnosis by randomisation allocation. Table SIII. Characteristics of those with cytogenetic data at relapse by randomisation allocation. Table SIV. Characteristics of those with cytogenetic data at diagnosis and relapse by randomisation allocation. Table SV. Characteristics of those with cytogenetic data at diagnosis or relapse by randomisation allocation. Figure S1. Forest plot of the complete cytogenetic subgroup results for Response. The black squares and horizontal lines represent the odds of a ≥ VGPR response in the salvage ASCT arm compared to the weekly cyclophosphamide arm and the associated 95% confidence interval, p(het) represents the p‐value from the likelihood ratio test assessing heterogeneity of treatment effect between subgroups. Figure S2. Kaplan‐Meier curve for TTP by whether MYC was normal or rearranged at first relapse in patients randomised to (a) salvage ASCT and (b) weekly cyclophosphamide. Figure S3. Kaplan‐Meier curve for OS by whether MYC was normal or rearranged at first relapse in patients randomised to (a) salvage ASCT and (b) weekly cyclophosphamide. Figure S4. Kaplan‐Meier curve for OS with estimated 95% confidence intervals by randomised treatment assuming no treatment effect in either arm i.e. assuming neither group received an ASCT at any time point. [file BJH-185-450-s001.docx]

**Supplementary Materials**

**Supplementary Methods**

**Randomisation and Masking**

Following PAD (bortezomib, doxorubicin and dexamethasone) re-induction treatment and, where applicable, peripheral blood stem-cell mobilisation and harvesting (PBSC) eligible patients were randomized, between a second (salvage) ASCT or a non-transplant consolidation (NTC) strategy using weekly oral cyclophosphamide (weekly cyclophosphamide) on a 1:1 stratified randomisation by length of first remission (<18, 18–24, and >24 months) and response to re-induction treatment (stable disease or ≥partial response).

**Procedures**

The trial consisted of two treatment periods, re-induction treatment with PAD therapy and consolidation treatment with either weekly oral cyclophosphamide or high dose melphalan and an ASCT dependent on their randomisation allocation. The re-induction treatment consisted of sequential 21 day cycles of intravenous bortezomib 1·3 mg/m^2^ on days 1, 4, 8, and 11; intravenous doxorubicin 9 mg/m^2^ per day on days 1–4; and oral dexamethasone 40 mg/day on days 1–4, 8–11, and on days 15–18 during cycle 1 and days 1–4 during cycles 2–4), with supportive care as per local institutional protocols (aciclovir, co-trimoxazole, and a proton-pump inhibitor). Consolidation therapy consisted of either a single infusion of intravenous melphalan 200 mg/m^2^ followed by ASCT after 24–48 hours, or oral cyclophosphamide 400 mg/m^2^ per week for 12 weeks.

**Supplementary Tables**

**Table SI: Baseline demographics of patients with FISH cytogenetic analysis at trial registration (first relapse) and those not**

|  | **Yes**  **(N=149)** | **No**  **(N=148)** | **Registration (N=297)** | **Yes**  **(N=88)** | **No**  **(N=86)** | **Randomisation (N=174)** |
| --- | --- | --- | --- | --- | --- | --- |
| **Age at baseline** |  |  |  |  |  |  |
| Median (IQR) | 61 (56, 65) | 61 (55, 65) | 61 (55, 65) | 61 (56, 64) | 62 (56, 65) | 61 (56. 65) |
| **Patients gender** |  |  |  |  |  |  |
| Male | 110 (73.8%) | 98 (66.2%) | 208 (70.0%) | 66 (75.0%) | 60 (69.8%) | 126 (72.4%) |
| Female | 39 (26.2%) | 50 (33.8%) | 89 (30.0%) | 22 (25.0%) | 26 (30.2%) | 48 (27.6%) |
| **Patients race** |  |  |  |  |  |  |
| White | 139 (93.3%) | 128 (86.5%) | 267 (89.9%) | 84 (95.5%) | 77 (89.5%) | 161 (92.5%) |
| Mixed - White and Black Caribbean | 0 (0.0%) | 1 (0.7%) | 1 (0.3%) | 0 (0.0%) | 0 (0.0%) | 0 (0.0%) |
| Asian - Indian | 0 (0.0%) | 3 (2.0%) | 3 (1.0%) | 0 (0.0%) | 3 (3.5%) | 3 (1.7%) |
| Asian - Pakistani | 0 (0.0%) | 1 (0.7%) | 1 (0.3%) | 0 (0.0%) | 1 (1.2%) | 1 (0.6%) |
| Asian - Bangladeshi | 0 (0.0%) | 1 (0.7%) | 1 (0.3%) | 0 (0.0%) | 0 (0.0%) | 0 (0.0%) |
| Other Asian background | 1 (0.7%) | 1 (0.7%) | 2 (0.7%) | 0 (0.0%) | 1 (1.2%) | 1 (0.6%) |
| Black - Caribbean | 4 (2.7%) | 4 (2.7%) | 8 (2.7%) | 1 (1.1%) | 2 (2.3%) | 3 (1.7%) |
| Black - African | 0 (0.0%) | 4 (2.7%) | 4 (1.3%) | 0 (0.0%) | 1 (1.2%) | 1 (0.6%) |
| Other Black background | 1 (0.7%) | 1 (0.7%) | 2 (0.7%) | 1 (1.1%) | 0 (0.0%) | 1 (0.6%) |
| Other ethnic group | 2 (1.3%) | 0 (0.0%) | 2 (0.7%) | 0 (0.0%) | 0 (0.0%) | 0 (0.0%) |
| Not stated | 2 (1.3%) | 1 (0.7%) | 3 (1.0%) | 2 (2.3%) | 1 (1.2%) | 3 (1.7%) |
| Missing | 0 (0.0%) | 3 (2.0%) | 3 (1.0%) | 0 (0.0%) | 0 (0.0%) | 0 (0.0%) |
| **Paraprotein type** |  |  |  |  |  |  |
| IgG | 99 (66.4%) | 91 (61.5%) | 190 (64.0%) | 62 (70.5%) | 55 (64.0%) | 117 (67.2%) |
| IgA | 31 (20.8%) | 24 (16.2%) | 55 (18.5%) | 16 (18.2%) | 15 (17.4%) | 31 (17.8%) |
| IgM | 0 (0.0%) | 1 (0.7%) | 1 (0.3%) | 0 (0.0%) | 1 (1.2%) | 1 (0.6%) |
| IgD | 2 (1.3%) | 0 (0.0%) | 2 (0.7%) | 1 (1.1%) | 0 (0.0%) | 1 (0.6%) |
| Light chain only | 11 (7.4%) | 17 (11.5%) | 28 (9.4%) | 5 (5.7%) | 9 (10.5%) | 14 (8.0%) |
| Non-secretor | 3 (2.0%) | 6 (4.1%) | 9 (3.0%) | 2 (2.3%) | 3 (3.5%) | 5 (2.9%) |
| Missing | 3 (2.0%) | 9 (6.1%) | 12 (4.0%) | 2 (2.3%) | 3 (3.5%) | 5 (2.9%) |
| **Light chain type** |  |  |  |  |  |  |
| Lambda | 48 (32.2%) | 34 (23.0%) | 82 (27.6%) | 25 (28.4%) | 20 (23.3%) | 45 (25.9%) |
| Kappa | 91 (61.1%) | 94 (63.5%) | 185 (62.3%) | 56 (63.6%) | 55 (64.0%) | 111 (63.8%) |
| Missing | 10 (6.7%) | 20 (13.5%) | 30 (10.1%) | 7 (8.0%) | 11 (12.8%) | 18 (10.3%) |
| **ISS at baseline** |  |  |  |  |  |  |
| I | 95 (63.8%) | 93 (62.8%) | 188 (63.3%) | 56 (63.6%) | 52 (60.5%) | 108 (62.1%) |
| II | 31 (20.8%) | 30 (20.3%) | 61 (20.5%) | 20 (22.7%) | 19 (22.1%) | 39 (22.4%) |
| III | 15 (10.1%) | 12 (8.1%) | 27 (9.1%) | 6 (6.8%) | 6 (7.0%) | 12 (6.9%) |
| Missing | 8 (5.4%) | 13 (8.8%) | 21 (7.1%) | 6 (6.8%) | 9 (10.5%) | 15 (8.6%) |
| **Randomisation treatment** |  |  |  |  |  |  |
| Salvage ASCT | N/A | N/A | N/A | 43 (48.9%) | 46 (53.5%) | 89 (51.1%) |
| Weekly Cyclophosphamide | N/A | N/A | N/A | 45 (51.1%) | 40 (46.5%) | 85 (48.9%) |
| **Previous treatment response length** |  |  |  |  |  |  |
| <18 months | N/A | N/A | N/A | 5 (5.7%) | 0 (0.0%) | 5 (2.9%) |
| 18 - 24 months | N/A | N/A | N/A | 24 (27.3%) | 17 (19.8%) | 41 (23.6%) |
| > 24 months | N/A | N/A | N/A | 59 (67.0%) | 69 (80.2%) | 128 (73.6%) |
| **Response to re-induction treatment** |  |  |  |  |  |  |
| SD | N/A | N/A | N/A | 9 (10.2%) | 3 (3.5%) | 12 (6.9%) |
| More than PR (PR, VGPR, CR or sCR) | N/A | N/A | N/A | 79 (89.8%) | 83 (96.5%) | 162 (93.1%) |
| **PBSC mobilisation and harvest given** |  |  |  |  |  |  |
| Yes | N/A | N/A | N/A | 29 (33.0%) | 41 (47.7%) | 70 (40.2%) |
| No | N/A | N/A | N/A | 58 (65.9%) | 41 (47.7%) | 99 (56.9%) |
| Missing Data | N/A | N/A | N/A | 1 (1.1%) | 4 (4.7%) | 5 (2.9%) |

**Table SII: Characteristics of those with cytogenetic data at diagnosis by randomisation allocation**

|  | **Salvage ASCT (N=20)** | **Weekly Cyclophosphamide (N=26)** | **Total (N=46)** |
| --- | --- | --- | --- |
| **Age at baseline** |  |  |  |
| Median (IQR) | 60 (53, 64) | 62 (51,65) | 61 (51, 65) |
| **Patients gender** |  |  |  |
| Male | 16 (80.0%) | 19 (73.1%) | 35 (76.1%) |
| Female | 4 (20.0%) | 7 (26.9%) | 11 (23.9%) |
| **Patients race** |  |  |  |
| White | 18 (90.0%) | 25 (96.2%) | 43 (93.5%) |
| Asian - Indian | 0 (0.0%) | 1 (3.8%) | 1 (2.2%) |
| Asian - Pakistani | 1 (5.0%) | 0 (0.0%) | 1 (2.2%) |
| Black - Caribbean | 1 (5.0%) | 0 (0.0%) | 1 (2.2%) |
| **Paraprotein type** |  |  |  |
| IgG | 12 (60.0%) | 22 (84.6%) | 34 (73.9%) |
| IgA | 4 (20.0%) | 2 (7.7%) | 6 (13.0%) |
| IgM | 1 (5.0%) | 0 (0.0%) | 1 (2.2%) |
| Light chain only | 1 (5.0%) | 1 (3.8%) | 2 (4.3%) |
| Non-secretor | 0 (0.0%) | 1 (3.8%) | 1 (2.2%) |
| Missing | 2 (10.0%) | 0 (0.0%) | 2 (4.3%) |
| **Light chain type** |  |  |  |
| Lambda | 8 (40.0%) | 3 (11.5%) | 11 (23.9%) |
| Kappa | 10 (50.0%) | 22 (84.6%) | 32 (69.6%) |
| Missing | 2 (10.0%) | 1 (3.8%) | 3 (6.5%) |
| **ISS at baseline** |  |  |  |
| I | 11 (55.0%) | 19 (73.1%) | 30 (65.2%) |
| II | 8 (40.0%) | 4 (15.4%) | 12 (26.1%) |
| III | 1 (5.0%) | 1 (3.8%) | 2 (4.3%) |
| Missing | 0 (0.0%) | 2 (7.7%) | 2 (4.3%) |
| **Previous treatment response length** |  |  |  |
| <18 months | 1 (5.0%) | 1 (3.8%) | 2 (4.3%) |
| 18 - 24 months | 4 (20.0%) | 7 (26.9%) | 11 (23.9%) |
| > 24 months | 15 (75.0%) | 18 (69.2%) | 33 (71.7%) |
| **Response to re-induction treatment** |  |  |  |
| SD | 1 (5.0%) | 1 (3.8%) | 2 (4.3%) |
| More than PR (PR, VGPR, CR or sCR) | 19 (95.0%) | 25 (96.2%) | 44 (95.7%) |
| **PBSC mobilisation and harvest given** |  |  |  |
| Yes | 8 (40.0%) | 8 (30.8%) | 16 (34.8%) |
| No | 12 (60.0%) | 18 (69.2%) | 30 (65.2%) |

**Table SIII: Characteristics of those with cytogenetic data at relapse by randomisation allocation**

|  | **Salvage ASCT (N=43)** | **Weekly Cyclophosphamide (N=45)** | **Total (N=88)** |
| --- | --- | --- | --- |
| **Age at baseline** |  |  |  |
| Median (IQR) | 60 (56, 63) | 61 (55, 65) | 61 (56, 64) |
| **Patients gender** |  |  |  |
| Male | 30 (69.8%) | 36 (80.0%) | 66 (75.0%) |
| Female | 13 (30.2%) | 9 (20.0%) | 22 (25.0%) |
| **Patients race** |  |  |  |
| White | 42 (97.7%) | 42 (93.3%) | 84 (95.5%) |
| Black - Caribbean | 0 (0.0%) | 1 (2.2%) | 1 (1.1%) |
| Other Black background | 0 (0.0%) | 1 (2.2%) | 1 (1.1%) |
| Not stated | 1 (2.3%) | 1 (2.2%) | 2 (2.3%) |
| **Paraprotein type** |  |  |  |
| IgG | 32 (74.4%) | 30 (66.7%) | 62 (70.5%) |
| IgA | 6 (14.0%) | 10 (22.2%) | 16 (18.2%) |
| IgD | 0 (0.0%) | 1 (2.2%) | 1 (1.1%) |
| Light chain only | 2 (4.7%) | 3 (6.7%) | 5 (5.7%) |
| Non-secretor | 1 (2.3%) | 1 (2.2%) | 2 (2.3%) |
| Missing | 2 (4.7%) | 0 (0.0%) | 2 (2.3%) |
| **Light chain type** |  |  |  |
| Lambda | 14 (32.6%) | 11 (24.4%) | 25 (28.4%) |
| Kappa | 24 (55.8%) | 32 (71.1%) | 56 (63.6%) |
| Missing | 5 (11.6%) | 2 (4.4%) | 7 (8.0%) |
| **ISS at baseline** |  |  |  |
| I | 30 (69.8%) | 26 (57.8%) | 56 (63.6%) |
| II | 7 (16.3%) | 13 (28.9%) | 20 (22.7%) |
| III | 4 (9.3%) | 2 (4.4%) | 6 (6.8%) |
| Missing | 2 (4.7%) | 4 (8.9%) | 6 (6.8%) |
| **Previous treatment response length** |  |  |  |
| <18 months | 3 (7.0%) | 2 (4.4%) | 5 (5.7%) |
| 18 - 24 months | 12 (27.9%) | 12 (26.7%) | 24 (27.3%) |
| > 24 months | 28 (65.1%) | 31 (68.9%) | 59 (67.0%) |
| **Response to re-induction treatment** |  |  |  |
| SD | 6 (14.0%) | 3 (6.7%) | 9 (10.2%) |
| More than PR (PR, VGPR, CR or sCR) | 37 (86.0%) | 42 (93.3%) | 79 (89.8%) |
| **PBSC mobilisation and harvest given** |  |  |  |
| Yes | 18 (41.9%) | 11 (24.4%) | 29 (33.0%) |
| No | 25 (58.1%) | 33 (73.3%) | 58 (65.9%) |
| Missing Data | 0 (0.0%) | 1 (2.2%) | 1 (1.1%) |

**Table SIV: Characteristics of those with cytogenetic data at diagnosis and relapse by randomisation allocation**

|  | **Salvage ASCT (N=13)** | **Weekly Cyclophosphamide (N=17)** | **Total (N=30)** |
| --- | --- | --- | --- |
| **Age at baseline** |  |  |  |
| Median (IQR) | 59 (56, 63) | 62 (54, 65) | 62 (54, 65) |
| **Patients gender** |  |  |  |
| Male | 12 (92.3%) | 13 (76.5%) | 25 (83.3%) |
| Female | 1 (7.7%) | 4 (23.5%) | 5 (16.7%) |
| **Patients race** |  |  |  |
| White | 13 (100.0%) | 17 (100.0%) | 30 (100.0%) |
| **Paraprotein type** |  |  |  |
| IgG | 8 (61.5%) | 14 (82.4%) | 22 (73.3%) |
| IgA | 4 (30.8%) | 2 (11.8%) | 6 (20.0%) |
| Light chain only | 0 (0.0%) | 1 (5.9%) | 1 (3.3%) |
| Missing | 1 (7.7%) | 0 (0.0%) | 1 (3.3%) |
| **Light chain type** |  |  |  |
| Lambda | 5 (38.5%) | 2 (11.8%) | 7 (23.3%) |
| Kappa | 7 (53.8%) | 15 (88.2%) | 22 (73.3%) |
| Missing | 1 (7.7%) | 0 (0.0%) | 1 (3.3%) |
| **ISS at baseline** |  |  |  |
| I | 7 (53.8%) | 11 (64.7%) | 18 (60.0%) |
| II | 6 (46.2%) | 3 (17.6%) | 9 (30.0%) |
| III | 0 (0.0%) | 1 (5.9%) | 1 (3.3%) |
| Missing | 0 (0.0%) | 2 (11.8%) | 2 (6.7%) |
| **Previous treatment response length** |  |  |  |
| <18 months | 1 (7.7%) | 1 (5.9%) | 2 (6.7%) |
| 18 - 24 months | 3 (23.1%) | 4 (23.5%) | 7 (23.3%) |
| > 24 months | 9 (69.2%) | 12 (70.6%) | 21 (70.0%) |
| **Response to re-induction treatment** |  |  |  |
| SD | 1 (7.7%) | 0 (0.0%) | 1 (3.3%) |
| More than PR (PR, VGPR, CR or sCR) | 12 (92.3%) | 17 (100.0%) | 29 (96.7%) |
| **PBSC mobilisation and harvest given** |  |  |  |
| Yes | 7 (53.8%) | 3 (17.6%) | 10 (33.3%) |
| No | 6 (46.2%) | 14 (82.4%) | 20 (66.7%) |

**Table SV: Characteristics of those with cytogenetic data at diagnosis or relapse by randomisation allocation**

|  | **Salvage ASCT (N=50)** | **Weekly Cyclophosphamide (N=54)** | **Total (N=104)** |
| --- | --- | --- | --- |
| **Age at baseline** |  |  |  |
| Median (IQR) | 60 (56, 63) | 60 (54, 65) | 60 (55, 64) |
| **Patients gender** |  |  |  |
| Male | 34 (68.0%) | 42 (77.8%) | 76 (73.1%) |
| Female | 16 (32.0%) | 12 (22.2%) | 28 (26.9%) |
| **Patients race** |  |  |  |
| White | 47 (94.0%) | 50 (92.6%) | 97 (93.3%) |
| Asian - Indian | 0 (0.0%) | 1 (1.9%) | 1 (1.0%) |
| Asian - Pakistani | 1 (2.0%) | 0 (0.0%) | 1 (1.0%) |
| Black - Caribbean | 1 (2.0%) | 1 (1.9%) | 2 (1.9%) |
| Other Black background | 0 (0.0%) | 1 (1.9%) | 1 (1.0%) |
| Not stated | 1 (2.0%) | 1 (1.9%) | 2 (1.9%) |
| **Paraprotein type** |  |  |  |
| IgG | 36 (72.0%) | 38 (70.4%) | 74 (71.2%) |
| IgA | 6 (12.0%) | 10 (18.5%) | 16 (15.4%) |
| IgM | 1 (2.0%) | 0 (0.0%) | 1 (1.0%) |
| IgD | 0 (0.0%) | 1 (1.9%) | 1 (1.0%) |
| Light chain only | 3 (6.0%) | 3 (5.6%) | 6 (5.8%) |
| Non-secretor | 1 (2.0%) | 2 (3.7%) | 3 (2.9%) |
| Missing | 3 (6.0%) | 0 (0.0%) | 3 (2.9%) |
| **Light chain type** |  |  |  |
| Lambda | 17 (34.0%) | 12 (22.2%) | 29 (27.9%) |
| Kappa | 27 (54.0%) | 39 (72.2%) | 66 (63.5%) |
| Missing | 6 (12.0%) | 3 (5.6%) | 9 (8.7%) |
| **ISS at baseline** |  |  |  |
| I | 34 (68.0%) | 34 (63.0%) | 68 (65.4%) |
| II | 9 (18.0%) | 14 (25.9%) | 23 (22.1%) |
| III | 5 (10.0%) | 2 (3.7%) | 7 (6.7%) |
| Missing | 2 (4.0%) | 4 (7.4%) | 6 (5.8%) |
| **Previous treatment response length** |  |  |  |
| <18 months | 3 (6.0%) | 2 (3.7%) | 5 (4.8%) |
| 18 - 24 months | 13 (26.0%) | 15 (27.8%) | 28 (26.9%) |
| > 24 months | 34 (68.0%) | 37 (68.5%) | 71 (68.3%) |
| **Response to re-induction treatment** |  |  |  |
| SD | 6 (12.0%) | 4 (7.4%) | 10 (9.6%) |
| More than PR (PR, VGPR, CR or sCR) | 44 (88.0%) | 50 (92.6%) | 94 (90.4%) |
| **PBSC mobilisation and harvest given** |  |  |  |
| Yes | 19 (38.0%) | 16 (29.6%) | 35 (33.7%) |
| No | 31 (62.0%) | 37 (68.5%) | 68 (65.4%) |
| Missing Data | 0 (0.0%) | 1 (1.9%) | 1 (1.0%) |

**Figure S1: Forest plot of the complete cytogenetic subgroup results for Response. The black squares and horizontal lines represent the odds of a ≥ VGPR response in the salvage ASCT arm compared to the weekly cyclophosphamide arm and the associated 95% confidence interval, p(het) represents the p-value from the likelihood ratio test assessing heterogeneity of treatment effect between subgroups.**

**Figure S2: Kaplan-Meier curve for TTP by whether MYC was normal or rearranged at first relapse in patients randomised to (a) salvage ASCT and (b) weekly cyclophosphamide.**

A

**
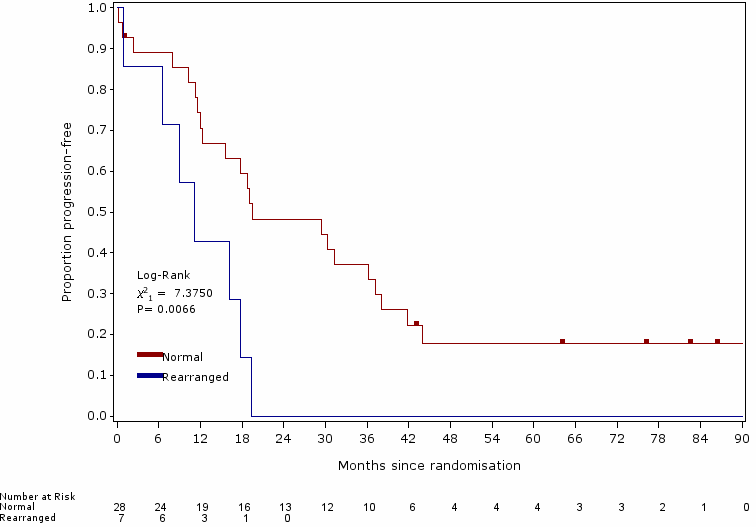
**

**
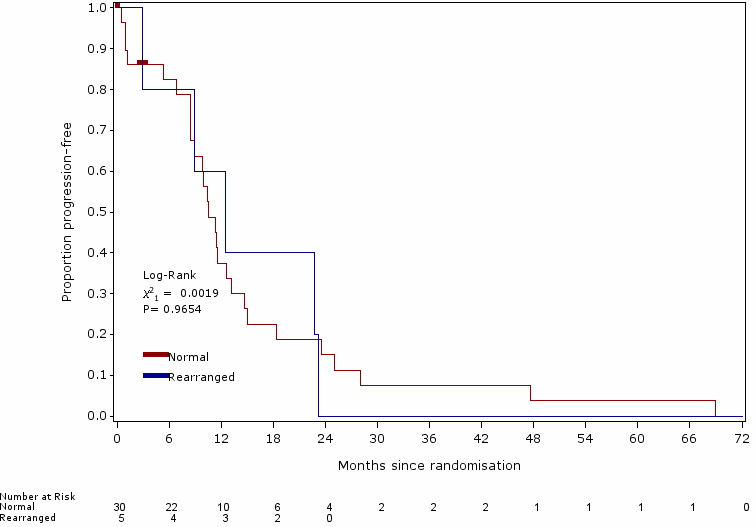
**

B

**
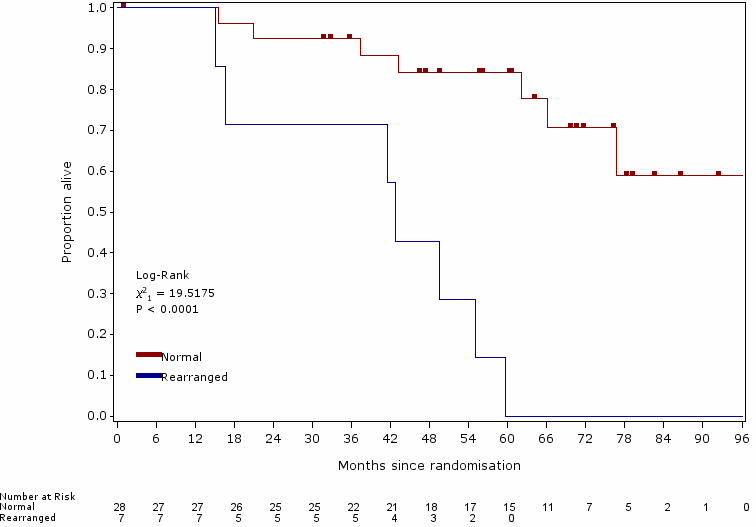
Figure S3: Kaplan-Meier curve for OS by whether MYC was normal or rearranged at first relapse in patients randomised to (a) salvage ASCT and (b) weekly cyclophosphamide.**

A

B

**
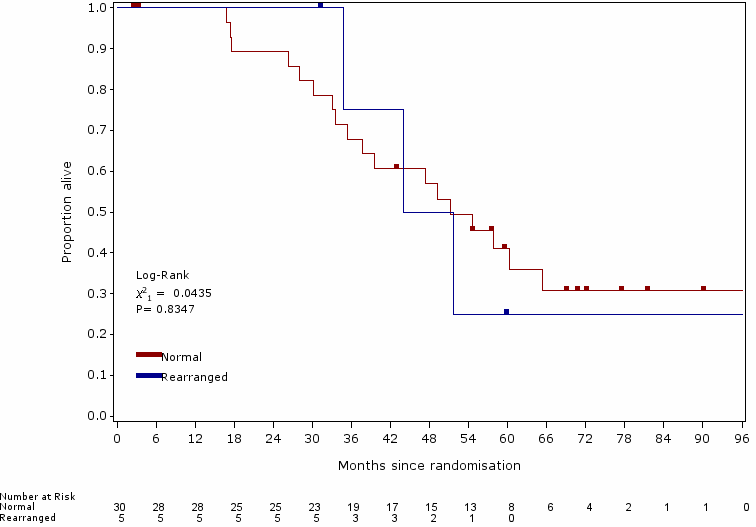
**

**Figure S4: Kaplan-Meier curve for OS with estimated 95% confidence intervals by randomised treatment assuming no treatment effect in either arm i.e. assuming neither group received an ASCT at any time point**

**Study Co-investigators**

In addition to the authors, the following investigators participated in the study:

| **Recruiting centre** | **Principal Investigator** | **Number of participants registered** |
| --- | --- | --- |
| Nottingham University Hospital | Dr Cathy Williams | 30 |
| University College London Hospital | Dr Kwee Yong | 17 |
| Royal Hallamshire Hospital | Dr John Snowden | 14 |
| Leeds Teaching Hospitals | Prof Gordon Cook | 14 |
| Derriford Hospital | Dr Hannah Hunter | 11 |
| Christie Hospital | Dr Jim Cavet | 10 |
| St. Bartholomew's Hospital | Dr Heather Oakervee | 10 |
| Bristol Haematology & Oncology Centre | Dr Jenny Bird | 9 |
| Birmingham Heartlands Hospital | Dr Guy Pratt | 8 |
| Gloucestershire Royal Hospital | Dr Sally Chown | 8 |
| Glan Clwyd | Dr Earnest Heartin | 7 |
| Manchester Royal Infirmary | Dr Eleni Tholouli | 7 |
| Addenbrookes Hospital | Dr Jenny Craig | 7 |
| Ipswich Hospital | Dr A J Ademokun | 7 |
| Royal Derby Hospital | Dr David Allotey | 7 |
| Castle Hill Hospital | Dr Haz Sayala | 7 |
| Medway Maritime Hospital | Dr Vivienne Andrews | 6 |
| Southampton University Hospital | Dr Matthew Jenner | 6 |
| Guy’s & St Thomas’ NHS Foundation Trust | Dr Majid Kazmi | 5 |
| Frenchay Hospital | Dr Alastair Whiteway | 5 |
| Singleton Hospital | Dr Hamdi Sati | 5 |
| Kings College Hospital | Prof Steve Schey | 5 |
| Leicester Royal Infirmary | Dr Claire Chapman | 5 |
| James Cook Hospital | Dr Angela Wood | 4 |
| St Helier & Epsom Hospitals | Dr Simon Stern | 4 |
| Queen Elizabeth Hospital, Birmingham | Dr Mark Cook | 4 |
| Aberdeen Royal Infirmary | Dr Jane Tighe | 4 |
| Colchester Hospital | Dr Gavin Campbell | 4 |
| Rotherham General Hospital | Dr Helen Barker | 4 |
| Beatson West of Scotland Cancer Centre | Dr Grant McQuaker | 4 |
| Belfast City Hospital | Dr Mary Drake | 4 |
| Ysbyty Gwynedd | Dr Melinda Hamilton | 3 |
| Stafford Hospital | Dr Paul Revell | 3 |
| Royal Berkshire NHS Foundation Trust | Dr Henri Grech | 3 |
| Chesterfield Royal Hospital | Dr Emma Welch | 3 |
| Doncaster Royal Infirmary | Dr Youssef Sorour | 3 |
| St Georges Hospital | Dr Fenella Willis | 3 |
| Ninewells Hospital | Dr Duncan Gowans | 2 |
| Bradford Royal Infirmary | Dr Samuel Ackroyd | 2 |
| Crosshouse and Ayr Hospitals | Dr Julie Gillies | 2 |
| Norfolk & Norwich Hospital | Dr Martin Auger | 2 |
| Diana Princess of Wales Hospital | Dr Susan Levison‐Keating | 2 |
| Raigmore Hospital | Dr Peter Forsyth | 2 |
| Royal Devon & Exeter Hospital | Dr Malcolm Hamilton | 2 |
| Sandwell & West Birmingham Hospitals | Dr Farooq Wandroo | 2 |
| University Hospital Coventry | Dr Syed Bokhari | 2 |
| University Hospital of Wales, Cardiff | Dr Keith Wilson | 2 |
| Dorset County Hospital | Dr Akeel Moosa | 2 |
| Queens Hospital, Burton | Dr Hamayun Ahmed | 2 |
| Torbay Hospital | Dr Deborah Turner | 2 |
| Cheltenham General Hospital | Dr Sally Chown | 1 |
| The Great Western Hospital, Swindon | Dr Norbert Blesing | 1 |
| United Lincolnshire Hospitals | Dr Kandeepan Saravanamuttu | 1 |
| Peterborough District Hospital | Dr S Kumar Nagumantry | 1 |
| Salisbury Hospital | Dr Jonathan Cullis | 1 |
| Mid Yorkshire Hospitals NHS Trust | Dr John Ashcroft | 1 |
| Russells Hall Hospital | Dr Savio Fernandes | 1 |
| Countess of Chester Hospital | Dr Salaheddin Tueger | 1 |
| Royal Oldham Hospital | Dr Vivek Sen | 1 |
| Warwick Hospital | Dr Anton Borg | 1 |
| Royal Bournemouth Hospital | Dr Helen McCarthy | 1 |
